# Assessment of transfemoral amputees using a passive microprocessor-controlled knee versus an active powered microprocessor-controlled knee for level walking

**DOI:** 10.1186/s12938-016-0287-6

**Published:** 2016-12-19

**Authors:** Veerle Creylman, Ingrid Knippels, Paul Janssen, Evelyne Biesbrouck, Knut Lechler, Louis Peeraer

**Affiliations:** 1 Mobilab, Thomas More Kempen, Kleinhoefstraat 4, 2440 Geel, Belgium; 2 Ossur hf, R&D Medical Office, Grjothals 5-7, 110 Reykjavik, Iceland; 3 Department of Rehabilitation Sciences, KU Leuven, Tervuursevest 101, 3001 Louvain, Belgium

**Keywords:** Active prosthesis, Transfemoral amputee, Prosthesis, Artificial limbs, Knee, Patient satisfaction, Rehabilitation

## Abstract

**Background:**

In transfemoral (TF) amputees, the forward propulsion of the prosthetic leg in swing has to be mainly carried out by hip muscles. With hip strength being the strongest predictor to ambulation ability, an active powered knee joint could have a positive influence, lowering hip loading and contributing to ambulation mobility. To assess this, gait of four TF amputees was measured for level walking, first while using a passive microprocessor-controlled prosthetic knee (P-MPK), subsequently while using an active powered microprocessor-controlled prosthetic knee (A-MPK). Furthermore, to assess long-term effects of the use of an A-MPK, a 4-weeks follow-up case study was performed.

**Methods:**

The kinetics and kinematics of the gait of four TF amputees were assessed while walking with subsequently the P-MPK and the A-MPK. For one amputee, a follow-up study was performed: he used the A-MPK for 4 weeks, his gait was measured weekly.

**Results:**

The range of motion of the knee was higher on both the prosthetic and the sound leg in the A-MPK compared to the P-MPK. Maximum hip torque (HT) during early stance increased for the prosthetic leg and decreased for the sound leg with the A-MPK compared to the P-MPK. During late stance, the maximum HT decreased for the prosthetic leg. The difference between prosthetic and sound leg for HT disappeared when using the A-MPK. Also, an increase in stance phase duration was observed. The follow-up study showed an increase in confidence with the A-MPK over time.

**Conclusions:**

Results suggested that, partially due to an induced knee flexion during stance, HT can be diminished when walking with the A-MPK compared to the P-MPK. The single case follow-up study showed positive trends indicating that an adaptation time is beneficial for the A-MPK.

## Background

Approximately 200–500 million major amputations are performed each year worldwide. Amputations of the lower extremities account for approximately 85% of those [1]. As a consequence of the absence of a natural limb, subjects with a unilateral transfemoral (TF) amputation often have asymmetries during gait [2–6]. Previous studies showed a longer stance phase and higher ankle, knee and hip joint moments and vertical ground reaction forces (GRFs) in the sound compared to the amputated limb and compared to able bodied subjects [5, 7]. Furthermore, it was shown that these factors increase the risk for injuries such as osteoarthritis, joint degeneration and low-back pain in TF amputees [5, 7–13].

To have a good prosthetic knee function is crucial for TF amputees. Efforts to replace knee function with mechanical devices have been met with varying degrees of success. Previous studies reported that there is a correlation between technological advances in prosthetic knee design and improved gait dynamics [14, 15]. For example, decreased frequency of falls and stumbles and increased user satisfaction have been demonstrated with the use of microprocessor-controlled prosthetic knee (MPK) units compared with mechanical devices [16, 17]. An example demonstrating improved clinical outcome of such a MPK is the Rheo Knee, a passive MPK (P-MPK, Össur, Reykjavik, Iceland) [15, 18].

Prosthetic knee joints are in general “passive” devices which give support in stance with the help of different mechanisms like e.g. damping, friction of a hinge axle or a polycentric geometry, but they do not generate power to assist the subject. An obvious solution to regain function and to lower proximal compensation mechanisms such as increased loading of the hip could be a prosthesis which is capable of generating power to restore concentric muscle activity.

Recently, this has been implemented in the Power Knee, the world’s first active microprocessor-controlled prosthetic knee (A-MPK, Össur, Reykjavik, Iceland) for TF amputees. While both the P-MPKs such as the Rheo Knee and the A-MPKs such as the Power Knee are microprocessor-controlled, they differ in that a P-MPK can only provide resistance, hence restoring eccentric muscle activity. On the contrary, an A-MPK provides resistance as well as active torque, therefore restoring both eccentric and concentric muscle activity around the knee by an electric motor. Thereby, A-MPKs such as the Power Knee, actively flex into swing phase and provide a gait speed dependent pre-flexed positioning (4–12 °) at initial contact via active extension at the end of swing. This pre-flexed positioning allows for a physiological stance phase knee flexion. To this day, most prosthetic knees still lack this knee flexion during stance phase [19–21]. In the A-MPK, active stance phase knee flexion is promoted in loading response to aid the extensor muscle activity of the gluteus. The gluteus muscle contracts in amputee gait from initial contact to mid-stance for pelvic control and propulsion of the body over the prosthetic foot to compensate for the lack of plantar flexors [22].

The aim of this study was to determine the difference between the P-MPK and the A-MPK in terms of walking speed (WS), stance phase duration (SPD), range of motion of the knee during loading response (KR) and hip torque (HT) in both sound and prosthetic limb during level walking. We hypothesized that the A-MPK will induce knee flexion during stance phase and that the HT will be lower from initial use of the A-MPK compared to when the P-MPK is used. Furthermore, we assessed whether these differences increase or decrease when subjects use the novel A-MPK for a longer time with a case study.

## Methods

### Subjects

Four male subjects (age = 51 ± 13 years; weight = 86.5 ± 2.3 kg) with a TF amputation participated in the study. Two of the subjects had an amputation of the right lower limb, two of the left. All subjects had been using the P-MPK for more than a year prior to this study, in combination with an ischial containment socket. The sockets used in daily life were also used during the measurements. Similar, the same prosthetic foot was used in both daily life and during the measurements; Subjects 1 and 3 used an energy storing and return (ESAR) foot with a vertical shock and rotation adapter (XC Rotate), while subject 2 was equipped with another ESAR foot (Vari-Flex XC). Subject 4 used an ESAR foot with a vertical shock as well as a rotation adapter (Re-Flex Rotate) (All feet from Össur hf, Reykjavik, Iceland). Subjects used their own shoes and provided written informed consent. The study was approved by the Medical Ethics Committee of UZ Leuven.

### Design

Data was collected for the four subjects, first while wearing their own prosthesis equipped with the P-MPK. Subsequently the A-MPK was fit and adjusted by a certified prosthetist and orthotist (CPO) according to manufacturer’s guidelines. Subjects were allowed a time of at least 30 min to adapt to the A-MPK. Afterwards, measurements with the A-MPK were conducted.

For one subject (Re-Flex Rotate ESAR foot, age 48), a follow-up study was conducted. This subject was instructed to use the A-MPK for 4 weeks, while each week his gait was measured according to the protocol.

### Protocol

Measurements were performed in a clinical gait lab on a walkway of 27 m. Active markers were attached on both sound and prosthetic limb using a specialized wand system provided by Charnwood Dynamics Ltd. [23]. Kinematic data were collected with these markers using the CODAMOTION system (Charnwood Dynamics Ltd., UK) with four CX1 cameras, measuring at 200 Hz. A force plate of 1 m long (RSScan, Olen, Belgium) was embedded in the walkway, measuring at 200 Hz, synchronized with the camera system. Subjects were asked to walk at a self-selected walking speed (SSWS). For each condition, five measurements of subsequently prosthetic and sound limb on the force plate were performed.

### Outcome measures

Spatial and temporal gait parameters being walking speed (WS) and stance phase duration (SPD) of both sound (SL) and prosthetic limb (PL) were calculated for each subject and each condition. Also, the gait asymmetry factor (ASF) was calculated for both knees. This is defined for the stance phase duration as follows:

$$ASF = \frac{(S - P)}{0.5\;(S + P)}.100$$

with S and P indicating values from the sound and prosthetic limb [24]. For both sound and prosthetic limb, maximum HT during stance phase in both flexion (at early stance) and extension (at late stance) was calculated for each condition, as well as the KR during loading response.

### Questionnaire

To assess the perception of the changes over time while wearing the A-MPK, in the case study at the end of each set of measurements a survey was conducted by the CPO in which the subject was asked about his comfort with the prosthesis on a visual analog scale (VAS). The following questions were asked to the subject:

- How satisfied are you with your prosthesis?
  extremely dissatisfied (1)–extremely dissatisfied (10)
- Rate the weight of your prosthesis? (10 = optimal weight; <10 = too low or too high)
- How much weight do you put on your prosthetic leg? (10 = equal load; <10 = more or less than non-prosthetic leg)
- Rate how much energy it took to walk 20 m with your prosthesis. (10 = none at all; 1 = completely exhausting)
- How is the feeling of the prosthesis on your residual limb? (10 = best possible; 1 = worst possible)
- Rate how satisfied you have been with how you are walking. (10 = extremely satisfied; 1 = extremely dissatisfied)

## Results

### P-MPK vs. A-MPK for four subjects

**Fig. 1:**
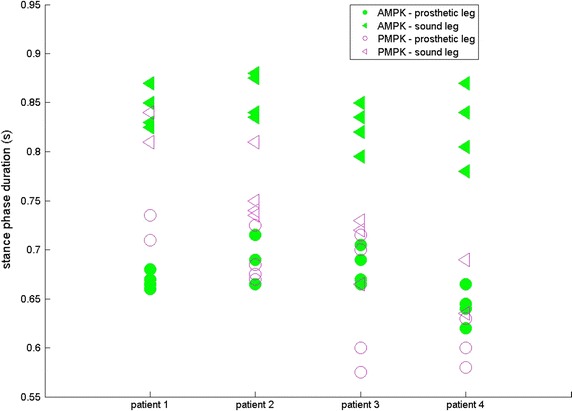
Results of the stance phase duration for four patients walking with the P-AMPK and the A-AMPK (s)

**Fig. 2:**
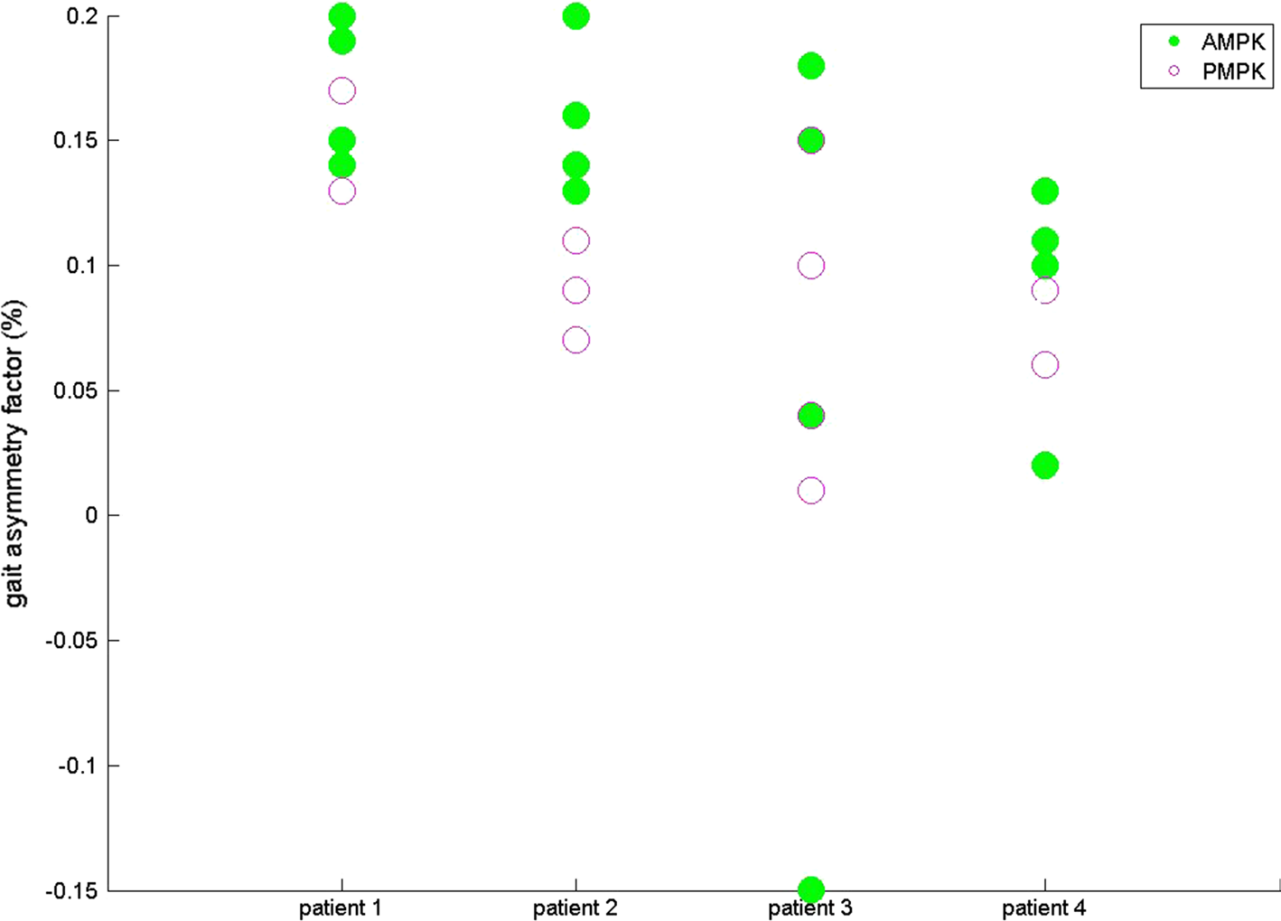
Results of the asymmetry factor of stance phase duration for four patients walking with the P-AMPK and the A-AMPK (m/s). A positive ASF indicates a longer stance phase duration of the SL compared to PL

**Fig. 3:**
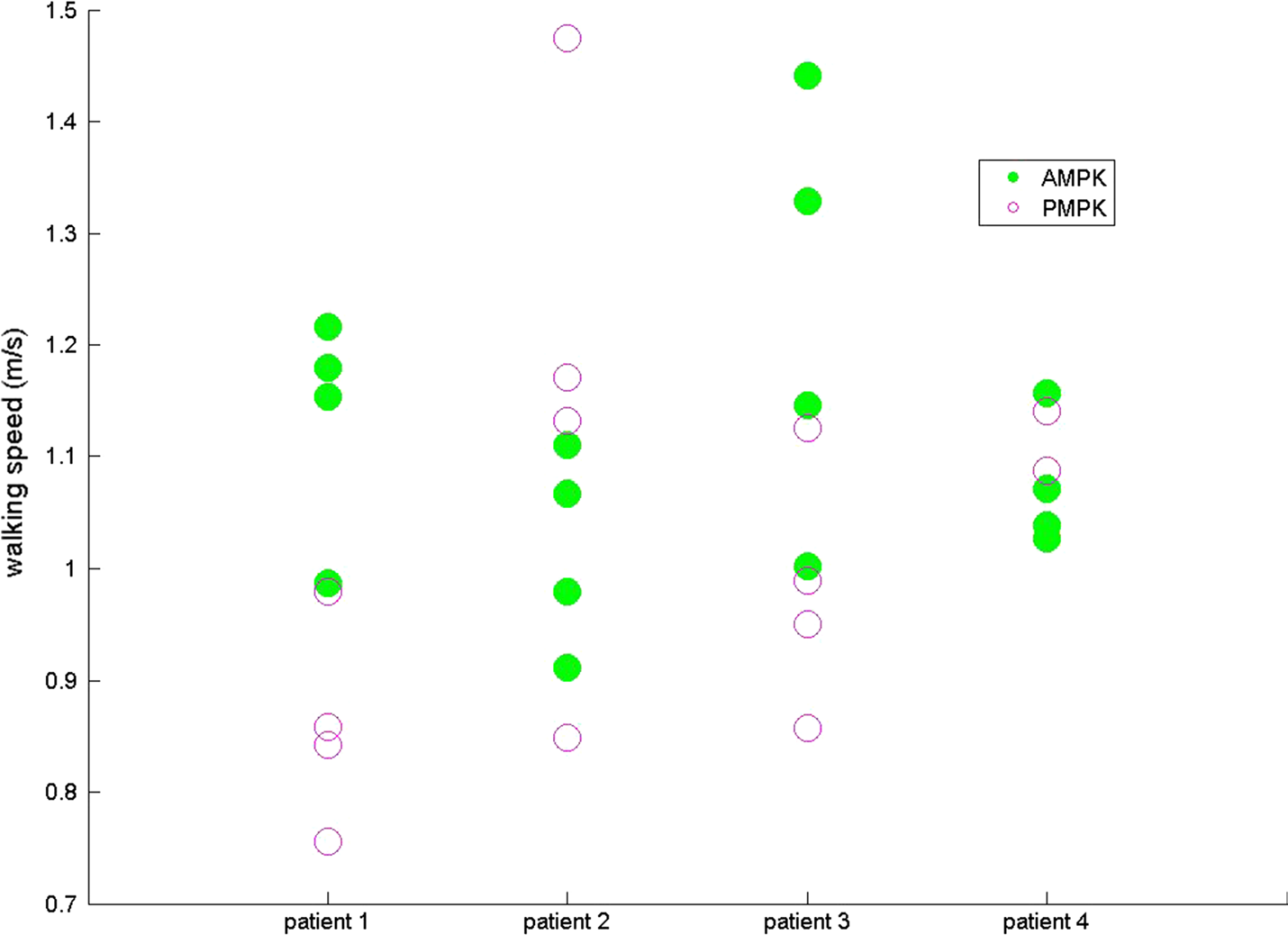
Results of the walking speed for four patients walking with the P-AMPK and the A-AMPK (m/s)

**Fig. 4:**
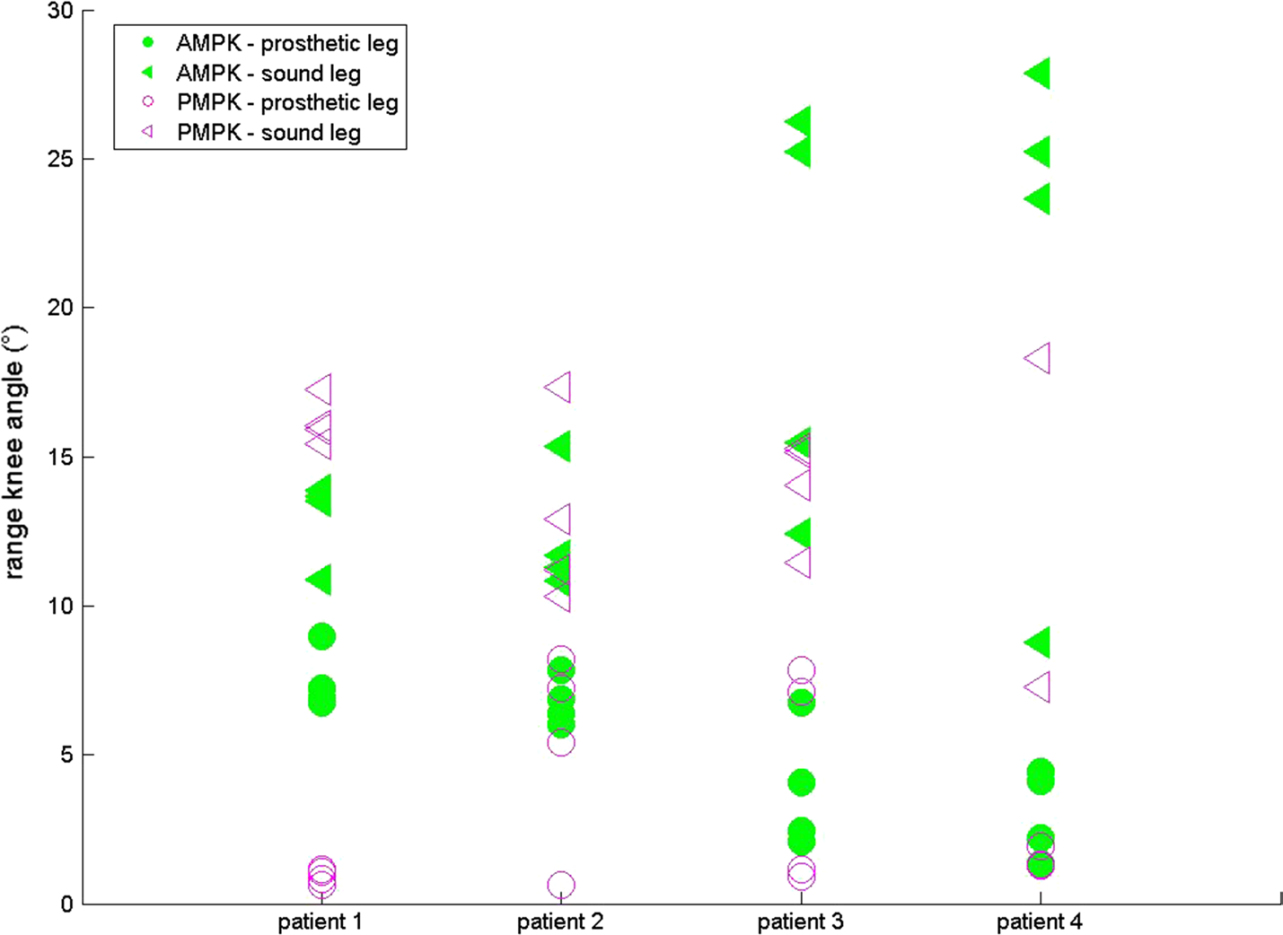
Results of the knee range during loading response for four patients walking with the P-AMPK and the A-AMPK (°)

**Fig. 5:**
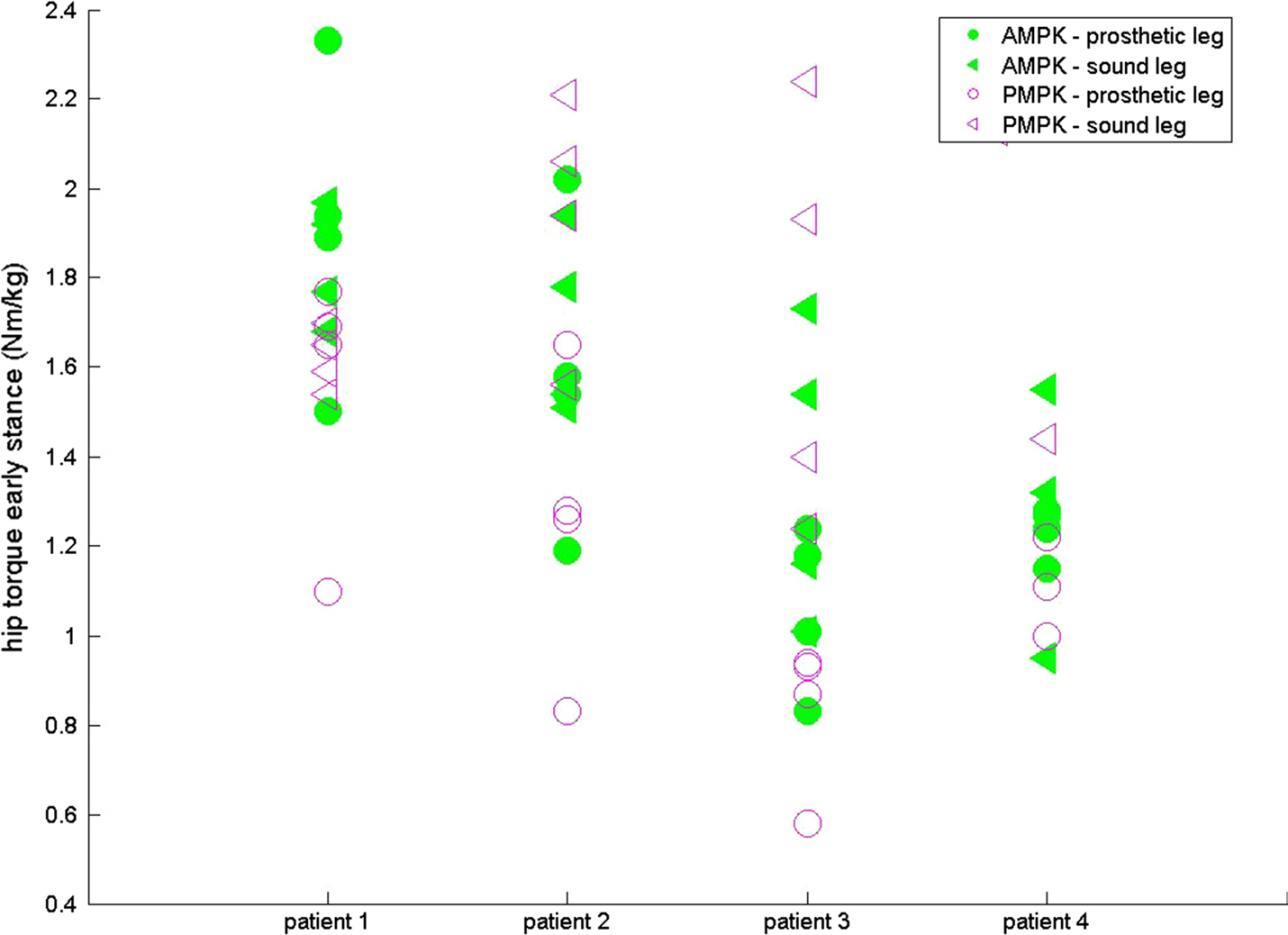
Results of the maximum hip torque during early stance for four patients walking with the P-AMPK and the A-AMPK (Nm/kg)

**Fig. 6:**
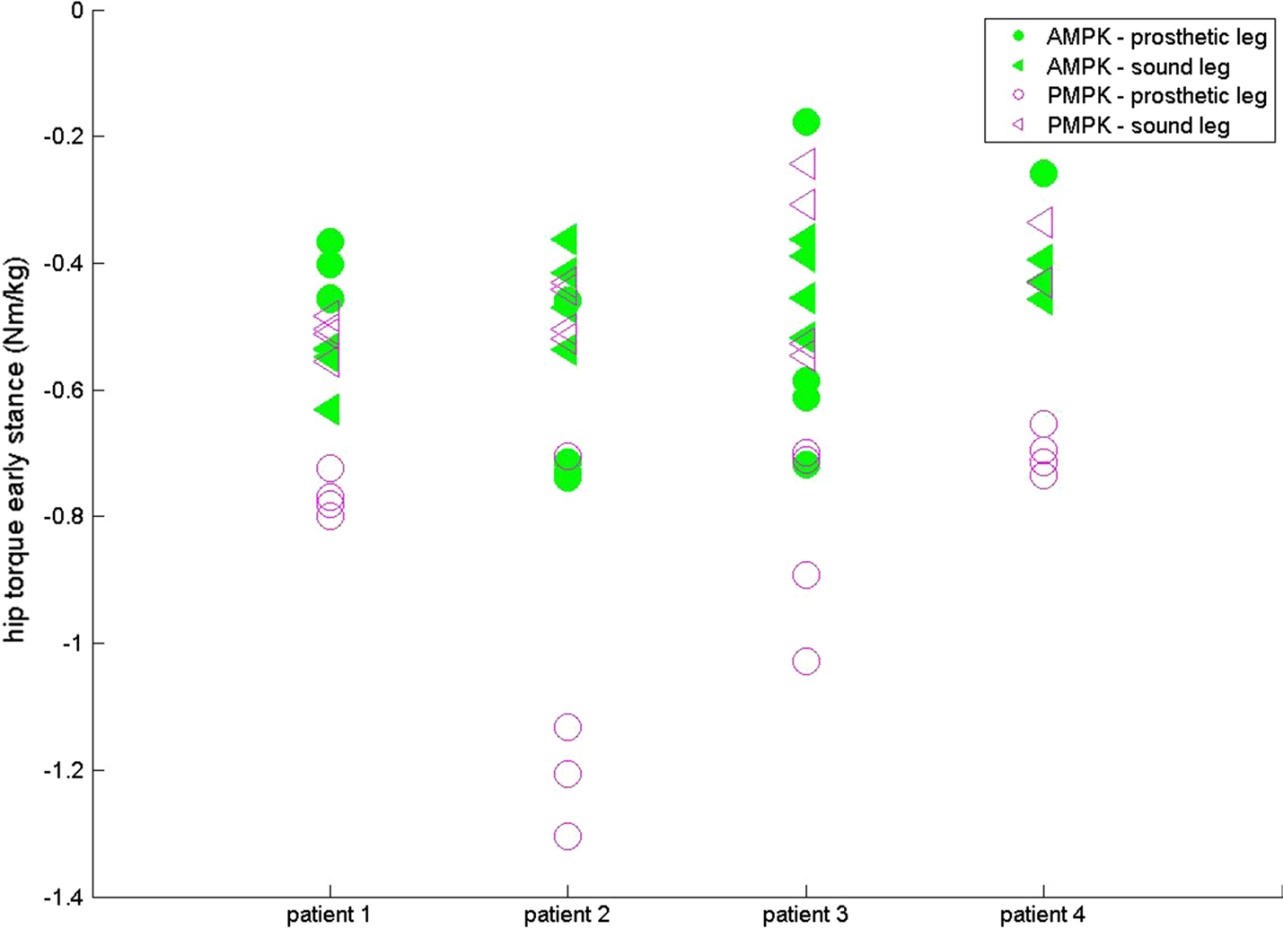
Results of the maximum hip torque during late stance for four patients walking with the P-AMPK and the A-AMPK (Nm/kg)

For four subjects, results are given for both sound (SL) and prosthetic leg (PL) for SPD (s—Fig. 1), ASF (Fig. 2), WS (m/s—Fig. 3), KR during loading response (°—Fig. 4) and maximum HT (Nm/kg) during both early (Fig. 5) and late stance (Fig. 6) for P-MPK and A-MPK.

### Follow-up study: one subject walking with A-MPK for 4 weeks

**Fig. 7:**
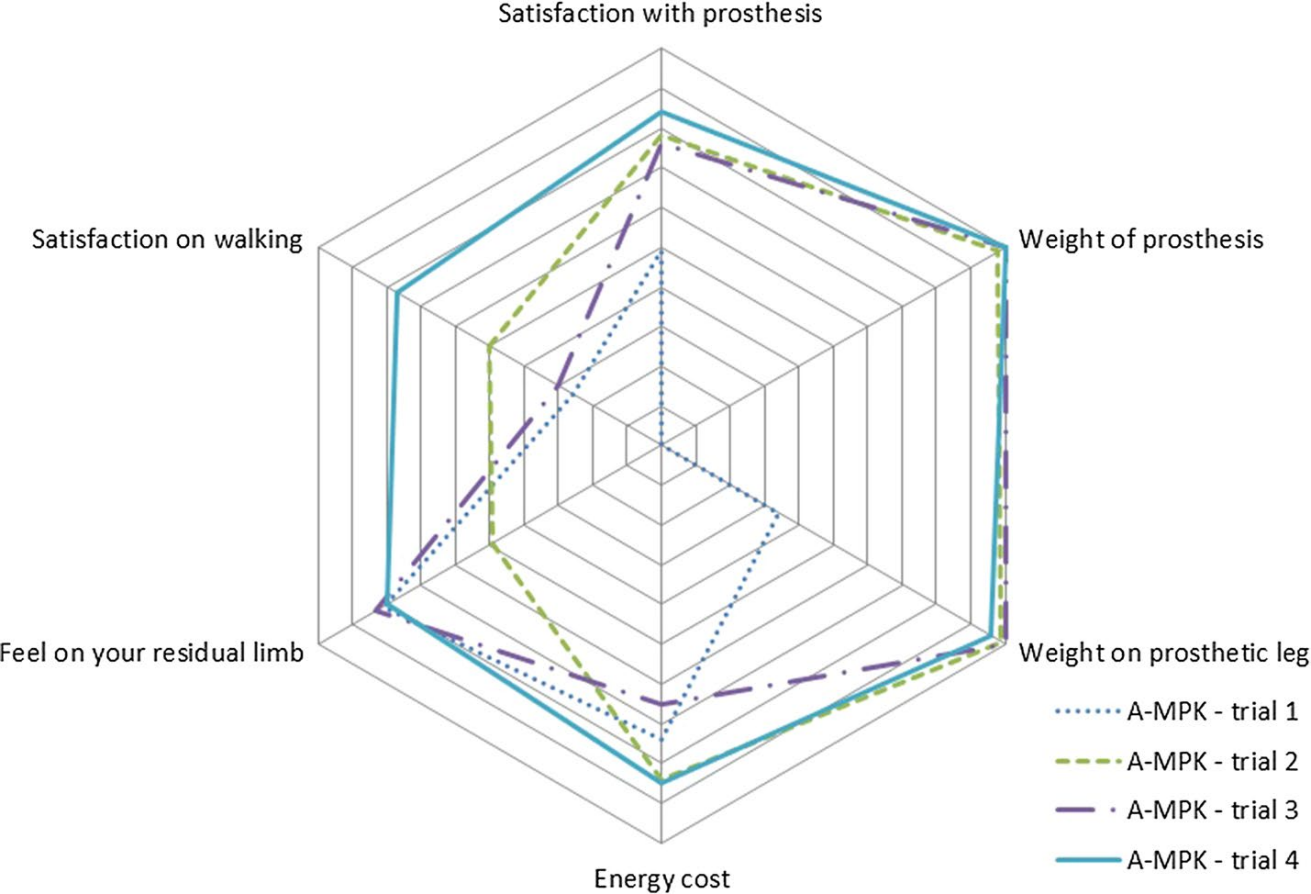
Results of the questionnaire: satisfaction of one patient walking with the A-MPK for four subsequent trials with a time interval of 1 week. The *middle* of the diagram represents 0 for low satisfaction, the *outer border* 10, for high satisfaction

For KR of the PL an increasing trend was seen during the first 3 weeks. For the SL, this trend was decreasing for 4 weeks. Maximum HT during late stance showed a decreasing trend over 4 weeks, for as well the PL as the SL. All other parameters did not show clear trends (Table 1). The results of the questionnaire of the four trials are presented in Fig. 7. The increasing area shows an increasing satisfaction and confidence with the A-MPK during the 4 week study.

**Table 1:** Results follow-up study, one subject measured weekly for 4 weeks while walking with the A-MPK

	Week 1		Week 2		Week 3		Week 4	
	PL	SL	PL	SL	PL	SL	PL	SL
SPD (s)	0.67 (0.01)	0.84 (0.02)	0.69 (0.02)	0.84 (0.03)	0.66 (0.01)	0.82 (0.03)	0.65 (0.02)	0.90 (0.02)
ASF	0.23 (0.01)		0.19 (0.04)		0.22 (0.04)		0.31 (0.03)	
KR (°)	3.35 (0.43)	14.66 (1.38)	3.69 (0.80)	13.08 (1.62)	4.03 (0.60)	12.51 (1.94)	3.88 (0.74)	11.32 (1.42)
Max HT early stance (Nm/kg)	2.42 (0.17)	2.04 (0.18)	1.81 (0.14)	1.93 (0.12)	1.64 (0.52)	2.12 (0.32)	1.84 (0.4)	1.65 (0.13)
Max HT late stance (Nm/kg)	0.85 (0.03)	0.52 (0.08)	0.72 (0.02)	0.51 (0.06)	0.65 (0.1)	0.22 (0.05)	0.49 (0.03)	0.49 (0.07)

Mean (standard deviation) of the of stance phase duration (SPD, s), asymmetry factor of stance phase duration (ASF), walking speed (WS, m/s), knee range during loading response (KR, °) and hip torque (HT, Nm/kg) during both early and late stance, for one subject measured weekly for 4 weeks while walking with the A-MPK

## Discussion

The objective of this study was to determine the difference between a P-MPK, a passive microprocessor-controlled prosthetic knee joint and the A-MPK, an active knee with an electric motor. Compared to the P-MPK, the A-MPK controls each degree of motion and provides active torque through an electric motor while the P-MPK can only provide resistance through magnetorheologic actuation in shear mode. Differences between the two knees were assessed in terms of WS, SPD, KR during loading response and maximum HT at early and late stance in both SL and PL.

Previous studies showed that there is a gait asymmetry in SPD between SL and PL in TF amputees: SPD of the SL is longer compared to the PL [4, 5, 25]. Similar results were found in this study: for both P-MPK and A-MPK stance phase of the SL has a longer duration than stance phase of the PL, suggesting that most of the load is taken by the SL. This is also translated in the ASF of SPD: the positive value indicates a longer SPD of the SL. When comparing the SPD for walking with the P-MPK with the SPD for walking with the A-MPK, for the SL, for all four subjects the SPD is in general longer for the A-MPK; for the PL, for two out of four subjects the SPD is longer for the A-MPK, for one subject the SPD remains the same. Since in general the differences in the ASF were small, the increase in both SL and PL is expected to be of the same magnitude. The same can be said for the WS: no general trend comparing P-MPK with A-MPK was observed.

The difference between the A-MPK and the P-MPK is that the A-MPK actively flexes and extends during swing phase and by doing this, it provides a gait speed dependent pre-flexed positioning (4–12 °) at initial contact. This pre-flexed position allows for a physiological knee flexion during loading response. In the P-MPK, and in other conventional prosthetic knees reported in literature [19–21], KR during loading response is limited. To compare it with the A-MPK, we assessed the KR during loading response. Results suggest an increase in KR during loading response, indicating that the A-MPK actively restores knee flexion during stance phase. Although not measured here, this can have a positive effect of the energy storage in the prosthetic foot: by flexing the knee, the ESAR-foot will have a different foot–ground contact and has the ability to store more energy in the foot. This will result in an increased support by the foot during push-off. Also, it results in the decrease in HT during push-off—which is also observed when comparing the PL of the A-MPK with the PL of the P-MPK.

This decrease in HT at push off at the PL was expected, together with a decrease in HT at early stance which is also observed in both PL and SL, and for the PL during late stance. Furthermore, the difference between the HT in SL and PL seems to diminish while walking with the A-MPK. This indicates a more symmetric gait, which is beneficial for the amputee.

One of the limitations of this study is the limited adaptation time to the A-MPK: the subjects were used to daily walking with prosthesis with a P-MPK for over a year, while they were only provided with the A-MPK half an hour prior to measurements and adapting to a new prosthetic component may require a learning phase [26]. To evaluate the effects of the A-MPK on a longer term, a case-study was performed where one subject was asked to walk with the A-MPK during daily life for 4 weeks and measurements were performed weekly. Furthermore, his perception of the prosthesis was evaluated using a questionnaire. Results suggest that a certain time is needed to adapt to the A-MPK: for the first 3 weeks, the maximum HT during early stance decreased for PL and the maximum HT during late stance decreased for the SL. Furthermore, WS increased during these weeks. However, in the last week WS decreased again, with the HTs increasing. A possible explanation for this finding might be tiredness of the subject during these last measurements; this was reported by the subject.

Finally, results of the questionnaire indicate an increase in satisfaction with the prosthesis, week after week: while at the first measurement the subject was not confident loading his PL, and he was not comfortable with the weight of the new prosthesis, his satisfaction and confidence increased week after week, increasing the suspicion that a time is needed to adapt to the A-MPK.

This study has some further limitations. The first additional limitation is the small number of subjects: a larger study is necessary to evaluate both immediate and long term effects of the A-MPK compared to the P-MPK and to draw statistical significant conclusions. A second additional limitation is that all subjects walked with their own prosthetic foot: the type of foot can influence the kinematic results as well. To overcome these limitations, an extended study including more subjects all wearing an identical prosthetic foot is considered.

## Conclusions

The gait of four subjects while walking with a prosthetic knee that actively flexes and extends during swing phase in addition to the passive control of the knee during stance phase, was compared to their gait while walking with a passive controlled knee. Results suggested that, partially due to an induced knee flexion during stance, hip torque was diminished. Also, an increase in stance phase duration was observed. Furthermore, results of a single-case walking with the active knee for a longer time showed improvements indicating that an adaptation time is beneficial. Results are limited by the small number of subjects.

## References

[1] Gait analysis after amputation. http://emedicine.medscape.com/article/1237638-overview. Accessed 5 May 2016.

[2] Boonstra AM, Schrama J, Fidler V, Eisma WH (1994). The gait of unilateral transfemoral amputees. Scand J Rehabil Med.

[3] Chow DHK, Holmes AD, Lee CKL, Sin SW (2006). The effect of prosthesis alignment on the symmetry of gait in subjects with unilateral transtibial amputation. Prosthet Orthot Int.

[4] Jaegers SM, Arendzen JH, de Jongh HJ (1995). Prosthetic gait of unilateral transfemoral amputees: a kinematic study. Arch Phys Med Rehabil.

[5] Nolan L, Wit A, Dudziñski K, Lees A, Lake M, Wychowañski M (2003). Adjustments in gait symmetry with walking speed in trans-femoral and trans-tibial amputees. Gait Posture.

[6] Seroussi RE, Gitter A, Czerniecki JM, Weaver K (1996). Mechanical work adaptations of above-knee amputee ambulation. Arch Phys Med Rehabil.

[7] Nolan L, Lees A (2000). The functional demands on the intact limb during walking for active trans-femoral and trans-tibial amputees. Prosthet Orthot Int.

[8] Burke MJ, Roman V, Wright V (1978). Bone and joint changes in lower limb amputees. Ann Rheum Dis.

[9] Ehde DM, Smith DG, Czerniecki JM, Campbell KM, Malchow DM, Robinson LR (2001). Back pain as a secondary disability in persons with lower limb amputations. Arch Phys Med Rehabil.

[10] Lemaire ED, Fisher FR (1994). Osteoarthritis and elderly amputee gait. Arch Phys Med Rehabil.

[11] Morgenroth DC, Orendurff MS, Shakir A, Segal A, Shofer J, Czerniecki JM (2010). The relationship between lumbar spine kinematics during gait and low-back pain in transfemoral amputees. Am J Phys Med Rehabil.

[12] Melzer I, Yekutiel M, Sukenik S (2001). Comparative study of osteoarthritis of the contralateral knee joint of male amputees who do and do not play volleyball. J Rheumatol.

[13] Norvell DC, Czerniecki JM, Reiber GE, Maynard C, Pecoraro JA, Weiss NS (2005). The prevalence of knee pain and symptomatic knee osteoarthritis among veteran traumatic amputees and nonamputees. Arch Phys Med Rehabil.

[14] Chin T, Sawamura S, Shiba R, Oyabu H, Nagakura Y, Takase I, Machida K, Nakagawa A (2003). Effect of an intelligent prosthesis (IP) on the walking ability of young transfemoral amputees: comparison of IP users with able-bodied people. Am J Phys Med Rehabil.

[15] Johansson JL, Sherrill DM, Riley PO, Bonato P, Herr H (2005). A clinical comparison of variable-damping and mechanically passive prosthetic knee devices. Am J Phys Med Rehabil.

[16] Hafner BJ, Willingham LL, Buell NC, Allyn KJ, Smith DG (2007). Evaluation of function, performance, and preference as transfemoral amputees transition from mechanical to microprocessor control of the prosthetic knee. Arch Phys Med Rehabil.

[17] Kahle JT, Highsmith MJ, Hubbard SL (2008). Comparison of nonmicroprocessor knee mechanism versus C-leg on prosthesis evaluation questionnaire, stumbles, falls, walking tests, stair descent, and knee preference. J Rehabil Res Dev.

[18] Greitemann B (2011). Quality of life improvements with a microprocessor-controlled prosthetic knee-first experiences in a cohort study. MOT.

[19] Bellmann M, Schmalz T, Blumentritt S (2010). Comparative biomechanical analysis of current microprocessor-controlled prosthetic knee joints. Arch Phys Med Rehabil.

[20] Bellmann M, Schmalz T, Ludwigs E, Blumentritt S (2012). Immediate effects of a new microprocessor-controlled prosthetic knee joint: a comparative biomechanical evaluation. Arch Phys Med Rehabil.

[21] Boonstra AM, Schrama JM, Eisma WH, Hof AL, Fidler V (1996). Gait analysis of transfemoral amputee patients using prostheses with two different knee joints. Arch Phys Med Rehabil.

[22] Raya MA, Gailey RS, Fiebert IM, Roach KE (2010). Impairment variables predicting activity limitation in individuals with lower limb amputation. Prosthet Orthot Int.

[23] Monaghan K, Delahunt E, Caulfield B (2007). Increasing the number of gait trial recordings maximises intra-rater reliability of the CODA motion analysis system. Gait Posture.

[24] Schaarschmidt M, Lipfert SW, Meier-Gratz C, Scholle HC, Seyfarth A (2012). Functional gait asymmetry of unilateral transfemoral amputees. Hum Mov Sci.

[25] De Castro MP, Soares D, Mendes E, Machado L (2014). Plantar pressures and ground reaction forces during walking of individuals with unilateral transfemoral amputation. PMR.

[26] Schmalz T, Bellmann M, Proebsting E, Blumentritt S (2014). Effects of adaptation to a functionally new prosthetic lower-limb component: results of biomechanical tests immediately after fitting and after 3 months of use. J Prosthet Orthot.

